# AI Machine Learning Technique Characterizes Potential Markers of Depression in Two Animal Models of Depression

**DOI:** 10.3390/brainsci13050763

**Published:** 2023-05-05

**Authors:** Jing Zhang, Ran Zhang, Ying Peng, Jiye Aa, Guangji Wang

**Affiliations:** Key Lab of Drug Metabolism & Pharmacokinetics, State Key Laboratory of Natural Medicines, China Pharmaceutical University, Tongjiaxiang 24, Nanjing 210009, China; jingzhang@stu.cpu.edu.cn (J.Z.); ranzhang@stu.cpu.edu.cn (R.Z.); pengy2014@126.com (Y.P.); jiyea@cpu.edu.cn (J.A.)

**Keywords:** proteomics, machine learning, AP2B1, SRCN1

## Abstract

(1) Background: there is an urgent clinical need for rapid and effective antidepressants. (2) Methods: We employed proteomics to profile proteins in two animal models (*n* = 48) of Chronic Unpredictable Stress and Chronic Social Defeat Stress. Additionally, partial least squares projection to latent structure discriminant analysis and machine learning were used to distinguish the models and the healthy control, extract and select protein features and build biomarker panels for the identification of different mouse models of depression. (3) Results: The two depression models were significantly different from the healthy control, and there were common changes in proteins in the depression-related brain regions of the two models; i.e., SRCN1 was down-regulated in the dorsal raphe nucleus in both models of depression. Additionally, SYIM was up-regulated in the medial prefrontal cortex in the two depression models. Bioinformatics analysis suggested that perturbed proteins are involved in energy metabolism, nerve projection, etc. Further examination confirmed that the trends of feature proteins were consistent with mRNA expression levels. (4) Conclusions: To the best of our knowledge, this is the first study to probe new targets of depression in multiple brain regions of two typical models of depression, which could be targets worthy of study.

## 1. Introduction

Depression is a serious psychiatric disorder with a worldwide influence [1]. The main first-line clinical drugs are currently serotonin, noradrenaline reuptake inhibitors and other drugs that act on monoaminergic neurotransmitters [2]. The largest problem with these drugs is that they are ineffective for some patients. Furthermore, they exhibit problems, such as a slow onset of 3–4 weeks, toxicity and considerable side effects. Research on the use of antidepressants to target NMDA receptor inhibitors has recently become a hot topic, but its clinical applicability is still controversial due to its highly addictive nature. In addition, the pathogenesis of depression is still unclear, as is the therapeutic target. Therefore, it is necessary to explore the pathogenesis of depression and identify new specific bio-targets to develop more effective antidepressants.

Depression is a kind of psychological disease. Clinically, doctors mainly judge the depressive state of patients according to the international diagnostic standard PQH9, the Hamilton depression rating scale, etc. Due to the difficulty in sampling brain tissue in clinical research, especially the difficulty in selecting the brain tissue of depressed patients in a specific period, it is very important to conduct disease research on animal models. However, the clinical diagnosis of depression relies mainly on communication between doctors and patients, and the relevant international diagnostic criteria are difficult to apply to rodents. Therefore, researchers have designed a variety of different animal models of depression and evaluated them for surface validity, construct validity and predictive validity, with a focus on describing the reproduction of certain aspects of human depression. These range from the initial Tail Suspension Test (TST) and Forced Swim Test (FST)—two acute depression models that characterize desperate behavior—to the Learned Helplessness Depression Model (LH) (to foot shock, etc.), to the more recognized Chronic Social Defeat Stress Model (CSDS) and Chronic Unpredictable Stress (CUMS) [3] Model. At present, the most widely used and highly recognized models mainly include CUMS and CSDS. The former is a mild depression model that embodies depression-like behaviors, such as anhedonia and behavioral despair, and focuses on simulating depression caused by the various unpredictable stressful stimuli people experience in their lives [4]; the latter can be a good representation of major depression and focuses on simulating depression-like behaviors, such as social avoidance and behavioral despair caused by long-term social frustration [5]. In view of the different emphases on the representation of depressive behaviors in different models, we should not only distinguish the differences between healthy control groups and disease groups but we should also distinguish the commonalities and differences between different models so as to better identify the distinct targets of depression.

With the advancement of research technology, people have gradually begun to realize the importance of omics, spanning from the metagenome to the transcriptome, proteome and metabolome. People use high-throughput big data screening methods in different dimensions to explore differential changes in diseases. Among them, proteomics takes proteins as the research object and studies their characteristics on a large scale, including protein expression, post-translational modifications and protein interactions. As a mature omics technology, proteomics has been extensively applied in many research fields, such as neuroscience [6], tumors [7], cardiovascular system diseases [8], traditional Chinese medicine and natural products [9], etc., and, for the discovery of molecular biomarkers of diseases [10], research on mechanism pathways has also received numerous contributions. In addition, proteomics also has many applications in depression. For example, van Haeringen, Marije et al. used proteomics to study changes in protein clusters in patients with clinical depression in order to more effectively find universal biomarkers of depression [11]; Hailan Hu et al. also applied proteomics to discover abnormal firing of the lateral rigid nucleus in depression [12]. However, as mentioned above, there is still a gap between preclinical research and clinical research at this stage. Therefore, applying a reliable brain proteome research protocol to rodent depression models has a high reference value for clinical research on depression.

As a whole, the brain has close connections between its regions, and a slight change in one region will affect the others. The research of a single brain region is insufficient and makes it difficult to understand the occurrence and development of depression from a holistic perspective. Therefore, it is necessary to select multiple depression-related brain regions for target exploration. Among the many depression-related studies, the hippocampus has been shown to play an important role in driving the long-term effects of antidepressants [13], and the functional connections between the hippocampus and other brain regions are closely related to human fear and to learning and memory [14]. In addition, clinical studies have shown that the effective connectivity of reward-related medial prefrontal cortical (mPFC)–limbic circuits in the brains of depressed patients is impaired [15] and that the inhibition of GABAergic neurons in the mPFC is sufficient and necessary for a rapid antidepressant response [16]. Finally, the dorsal raphe nucleus (DRN) is the main source of forebrain serotonergic innervation and is strongly associated with depression [17]. In addition, scientific and reasonable statistical data analysis methods help us to more quickly and accurately eliminate interfering factors and determine real differential changes. The partial least squares projection to latent structure discriminant analysis (PLS-DA) is often used to deal with classification and discrimination problems. Similarly to Principal Component Analysis (PCA), it also uses the principle of partial least squares to optimize the classification of complex factors; the difference is that PLS-DA is a supervised analysis and PCA is an unsupervised analysis [18]. In addition, one of the perceived advantages of PLS-DA is that it has the ability to analyze highly collinear and noisy data [19]. Machine learning (ML) is used to find the most representative features through machine algorithms, excluding linear correlation factors, and plays an important role in the analysis of various clinical data [20,21].

Therefore, we aim to compare the differential proteins under different depression models in the hippocampus and the DRN as well as the mPFC and apply a variety of data processing methods. We hope to use the fewest differential proteins and the most significant model discrimination to distinguish disease groups from healthy control groups and to distinguish between different disease models.

## 2. Materials and Methods

### 2.1. Animals

Both 7-week-old male C57BL/6J mice and male CD-1 mice over 6 months old (retired breeders) were bought from Vital River Laboratories, Beijing, China. Before the experiments, all the mice lived adaptively over seven days under standard laboratory conditions, which included a 12 h light/dark cycle (light from 7 am to 7 pm) and constant temperature and humidity (temperature: 24 ± 1 °C and humidity: 50~60%). During this period, 5 C57BL/6J mice were housed per cage, and the CD-1 mice were housed singly due to their aggressiveness. Before and during the experiment, all food and water could be accessed freely, and the feed complied with the national standards (“GB/T14924.2-2001 Hygiene Standard for Experimental Animal Compound Feed” and “GB/T14924.3-2010 Laboratory Animal Compound Feed Nutritional Composition Standard”), provided by Qinglongshan Experimental Animal Breeding Plant. All animal experimental procedures were approved by the Animal Ethics Committee of China Pharmaceutical University and performed in accordance with the US National Institutes of Health Guidelines.

### 2.2. Experimental Design

Our entire experimental process can be roughly divided into four steps: (1) Establishment of animal models and acquisition of samples; (2) Non-targeted proteomic detection; (3) Analysis of proteomic data using principal component analysis (PCA) and machine learning; (4) Differential verification of the characteristic proteins obtained from the analysis at the mRNA level.

In the first step, we applied two recognized depression models of CUMS mouse depression model and CSDS mouse depression model. Based on the depressive behavioral characteristics of mice after modeling, behavioral tests, such as social interaction test, sucrose preference test, tail suspension test and forced swimming test, were used to evaluate whether the models were successful.

Step two: Based on the successful establishment of the model, we performed non-targeted proteomics detection. First, the tissues of the hippocampus, DRN and mPFC brain regions of mice were prepared into peptide samples for mass spectrometry detection through a series of operations, such as protein extraction, deformation, alkylation, reduction and enzyme cutting. Secondly, the same amount of each sample was mixed to obtain a QC sample. The QC sample was subjected to DDA scanning. The data were used to establish a mouse brain protein ion library and also to determine the variable window width in the SWATH detection method. Finally, each brain region sample of the mice was subjected to SWATH detection (DIA scanning), and the qualitative and quantitative data of the proteins (protein data matrix) were obtained based on the self-built ion library.

In the third step, we used SIMCA software to perform PLD-DA analysis and R studio to perform machine learning analysis for the protein data matrix obtained in the second step.

Finally, the peak area quantitative information of the feature proteins screened by machine learning was normalized and subjected to routine statistical analysis in GraphPad software. Furthermore, PCR was used to evaluate the differences in the feature proteins at the mRNA level, and routine statistical analysis was also performed using GraphPad.

### 2.3. Depression Animal Models

#### 2.3.1. Chronic Unpredictable Mild Stress Model (CUMS) 

CUMS procedure (as previously described [4]): Firstly, C57BL/6J mice were divided into two groups: healthy control group (*n* = 6) and CUMS model group (*n* = 12). During the 33-day modeling process, model mice were continuously exposed to a variety of mild stressors, including being housed singly (24 h), over-crowding (10 mice per cage for 24 h), no water or food (24 h), 45° tilted cage, wet litter or no litter, physical constraint (6–8 h), forced swimming in cold water or hot water (3–15 min) and tail suspension (5 min per time). Every day, 2–3 types of stressors were randomly assigned to mice. 

#### 2.3.2. Chronic Social Defeat Stress Model (CSDS)

CSDS procedure (as previously described [5]): Firstly, aggressive CD-1 mice were selected for tests over 3 consecutive days. Then, C57BL/6J mice were divided into two groups: healthy control group (*n* = 6) and CSDS model group (*n* = 24). Every day, we put a C57BL/6J mouse of model group into a CD-1 cage; if a CD-1 mouse attacked a C57BL/6J mouse within one minute, this CD-1 could be regarded as aggressive. In addition, if a CD-1 showed aggressiveness over all three days, this CD-1 could be used for the model. Subsequently, during a 10-day defeat period, each C57BL/6J mouse was exposed to a new aggressive CD-1 and subjected to physical attacks for 10 min.

### 2.4. Behavioral Assays

#### 2.4.1. Social Interaction Test (SI)

As described in previous work [5], C57Bl/6J mice were allowed to adjust to the light and temperature in a behavioral test room for more than 30 min before the evaluation. Firstly, a C57BL/6J mouse was placed in a 40 × 40 × 40 cm mine, and an empty net cage was placed in the center of the social area. The activity of the C57BL/6J mouse was recorded over 2.5 min. Then, the mouse was removed and the mine area cleaned. In the second period, a new CD-1 mouse was placed in the net cage, and then the same C57BL/6J mouse was placed in the mine and the activity of the C57BL/6J mouse was recorded over 2.5 min. The sociability index (SI) was calculated as the ratio of the time C57BL/6J mice entered the social area during the second period to the time they entered the social area during the first period. If the C57BL/6J mouse had an SI value <1, this mouse could be considered depressed.

#### 2.4.2. Sucrose Preference Test (SPT) 

The SPT method was described in previous research [22]: Firstly, before the experiment, mice were given a bottle of water and water containing 2% sucrose for 24 h, and, during this time, the position of the water bottle was changed every 12 h. Subsequently, after the C57BL/6J mice had been deprived of water for 12 h, the consumption of water and syrup was noted by weighing and recording the weight of the water bottle and syrup bottle before and after the experiment. Finally, the calculation for sucrose preference was syrup consumption/(water consumption + syrup consumption) × 100%.

#### 2.4.3. Tail Suspension Test (TST) 

During this test, the tails of C57BL/6J mice were taped and fixed in a tail suspension box with their heads about 20 cm above the ground. Meanwhile, the mice were visually isolated from each other, and the activities of these mice were recorded for 6 min. The duration of immobility in mice in the last 4 min was analyzed by 2 colleagues who were blinded to this experiment, with immobility defined as complete immobility or only slight limb movement.

#### 2.4.4. Forced Swim Test (FST)

The forced swimming experiment method was previously described in the literature [23]. Mice were placed in a glass container with a diameter of 12 cm and a height of 30 cm. The height of the water surface was 20 cm under natural light, and the temperature of the water was 25 °C. A video camera was used to record the activities of the mice in the container for 6 min, and, during this period, the mice were blinded to each other. After the experiment, other colleagues, who were completely unaware of the experiment, analyzed the activity of the mice and recorded the duration of immobility during the last 4 min. When mice were standing upright on the water with their front paws together and only performing the movements necessary to stay afloat, they were considered stationary.

### 2.5. Untargeted Proteomics 

#### 2.5.1. Protein Sample Preparation

Considering the results of behavioral assays, we s reserved CUMS healthy control (*n* = 3), CUMS model (*n* = 6), CSDS healthy control (*n* = 3), CSDS model (*n* = 12) for further proteomics analyze. The samples of mouse brain were processed as previously described [24]. After we performed behavioral assays, the mice were anesthetized via intraperitoneal injection of avertin (7.2 mg/20 g body weight). Next, we performed transcardiac perfusion with saline in mice to quickly clear the blood. The brains of the mice were removed within 2 min, and the hippocampus, DRN and mPFC were isolated with reference to the Allen Brain Atlas. They were immediately placed in liquid nitrogen for long-term storage at −80 °C. We took appropriately sized mouse brain tissue samples and added RIPA lysis buffer to extract the proteins. Then, according to the method provided in the instructions of the BSA kit, the protein content of the supernatant was detected. Based on the calculated protein concentration of each sample, a total amount of undiluted supernatant equivalent to 200 μg protein was added to an ultrafiltration tube. First, the protein was denatured with 8M urea solution. After centrifuging at 14,000× *g* for 10 min at 4 °C, the supernatants were discarded. The disulfide bonds were then reduced with 10 mM DTT (final concentration) at 60 °C for an hour. After the alkylation reaction had taken place, freshly prepared 20 mM iodoacetamide solution (final concentration) was added, and the solution was reacted at room temperature for 45 min away from light; trypsin was added at a ratio of 1:50, and proteolytic digestion was performed for 16 h. Finally, the digested samples were taken from the supernatant and transferred to an injection vial. Online separation and determination of digested brain specimen were performed using the SCIEX M5 MicroLC microfluidic liquid system coupled to an AB SCIEX TripleTOF 5600 for the performance of non-targeted proteomic assays.

#### 2.5.2. Project-Specific DDA Library Generation

A protein ion library was established for the purpose of accurately identifying and quantifying the proteins detected in the non-targeted proteomic analysis. The establishment of the ion library consisted of two main steps: (1) DDA scanning of the QC samples; (2) Construction of two different ion libraries using ProteinPilot software (AB SCIEX, America, version 5.0.1) to match the results of the QC samples with the mouse protein database (version UniProtKB/Swiss-Prot Jan 2022, about 17,137 sequences) (Supporting Table S6).

In the first step, the TripleTOF 5600 (AB SCIEX, Framingham, MA, USA) mass spectrometry detection system is used for non-targeted protein profiling in proteomics with data-dependent acquisition mode (DDA). The mass spectrometry parameters are as follows: MS first-level scanning range *m*/*z* 350–1250, MS/MS second-level scanning range *m*/*z* 100–1500; source gas parameters: Gas1 = 12 psi, Gas2 = 20 psi, Curtain Gas = 30 psi; positive ion scanning mode, ionization voltage (ISV) = 5500 V; ion source temperature = 330 °C; declustering potential (DP) = 80 V, IDA number: 40; rolling CE: enabled; CES: 5.

#### 2.5.3. SWATH Method Construction 

The raw data of QC sample were imported into PeakView software (AB SCIEX, America, version 2.2) to obtain ion distribution data. Then, we used an analysis form provided by AB SCIEX (SWATH Variable Window Calculator_V1 in Excel Table S1) to obtain a window distribution file in txt format (Supporting Table S2). The parameters were as follows: the target number of windows was set to 88, the lower *m*/*z* limit to 350, the upper *m*/*z* limit to 1250 and the minimum window width (Da) to 4. Finally, we imported the window distribution file into Analyst software to perform a SWATH scan and set the mass spectrometry parameters as follows: ISV = 5500 V, GS1 = 12 psi, GS2 = 20 psi, CUR = 30 psi, DP = 80 V, TEM = 330 °C, TOF MS: *m*/*z*: 350–1250 Da and accumulation time: 0.15 s. The SWATH analysis parameters were as follows: window: 95, *m*/*z*: 350~1250, accumulation time: 96 ms and high sensitivity.

#### 2.5.4. Sample SWATH Detection

Based on the SCIEX M5 MicroLC microfluidic system (AB SCIEX, USA), the samples were separated on a C18 column (Eksigent ChromXP C18, 300 μm × 15 cm, 3 μm, 120 Å) using a 90 min 0.1% formic acid–acetonitrile (B) and 0.1% formic acid–water (A) gradient, setting column temperature to 40 °C, flow rate to 5μL/min. The gradient elution conditions: 0–0.1 min, 5–8% B; 0.1–78 min, 8–35% B; 78–80 min, 35–80% B; 80–84 min, 80% B; 84–85 min, 80–5% B; 85–90 min, 5% B.

The LC was coupled to the TripleTOF 5600 (AB SCIEX, USA) mass spectrometry detection system equipped with the SWATH module; the processed mouse brain region protein samples were scanned by DIA scan mode. Subsequently, using PeakView software, we matched the SWATH detection results with the previously established DDA ion library data (set a 95% confidence interval) to obtain the relative quantitative information of the proteins (Supporting Table S3).

### 2.6. Data Analysis 

#### 2.6.1. Principal Component Analysis 

The exported protein peak area data matrix was subjected to multivariate statistical analysis using SIMCA-P 14 software (Umetrics, Umeå, Sweden). First, we used supervised Partial Least Squares Discriminant Analysis (PLS-DA) to analyze the separation between different groups; then, we used Orthometric Partial Least Squares Discriminant Analysis (OPLS-DA) to compare the differences between the two groups and search for differential proteins. In addition, the differential proteins were analyzed using the PPI protein interaction network with the help of the string database (https://string-db.org Version: 11.5). It is noteworthy that, prior to conducting formal analysis, we utilized Hotelling’s T2 (95%) in SIMCA to diagnose strong outliers within the sample, ensuring that all available data fall within the 95% confidence interval.

#### 2.6.2. Machine Learning 

To build biomarker panels for the identification of different mouse models of depression, we used machine learning models. In brief, the exported proteomics data were randomly divided into two groups: test group (*n* = 24) and a validation group (*n* = 24). For the identification of proteomics biomarkers, we used the caret package (version 6.0-93) on the testing group to separately select features for the hippocampus, DRN and mPFC brain regions. The data were preprocessed before selection, including removing strongly correlated variables and multicollinearity. Then, all the feature proteins of the three brain regions were used for further (depression) protein selection using LASSO logistic regression algorithms (glmnet package, version 4.1-4), with penalty parameter tuning conducted using 5-fold cross-validation. The depression feature protein was generated via a linear combination of selected features weighted by their respective coefficients. The discrimination performance of the established models was quantified in the validation group using the receiver operating characteristic curve and area under the curve (AUC) values (pROC package, version 1.18.0). All statistical analyses were performed using R software (version 4.1.1, www.r-project.org). Finally, we performed pathway enrichment analysis on these selected proteins in the UK biobank database.

### 2.7. Quantitative Real-Time PCR (qPCR)

The qPCR was performed as previously described [25], with certain modifications. The sequences of the forward and reverse primers were as follows: 5′-TTTTGTGGAAGGGAGTCATGGG-3′ and 5′-TCACTGCCAAGCAGGCTATC-3′ for the AP-2 complex subunit beta (AP2B1) gene, 5′-AGCGAGATGCGTTCATGGAC-3′ and 5′-AACTCCAGTAGTTTGGTTGCTG-3′ for SRC kinase signaling inhibitor 1 (SRCN1) gene, 5′- CAATCAACGTCCGGGTGAC -3′ and 5′- GCCAATCGTCTTTACCACCTGA -3′ for the Ezrin (EZRI) gene and 5′-GGCTGTATTCCCCTCCATCG-3′ and 5′-CCAGTTGGTAACAATGCCATGT-3′ for the internal control 𝛽-actin gene. 

### 2.8. Statistical Analysis

The differential protein peak areas were analyzed using GraphPad (version 14.0) for routine statistical analysis. One-way ANOVA analysis of variance was used, followed by Tukey’s multiple comparisons test to compare the differences among the three groups, and an adjusted *p* value < 0.05 was considered significant.

## 3. Results

### 3.1. Two Different Kinds of Animal Depression Models

**Figure 1:**
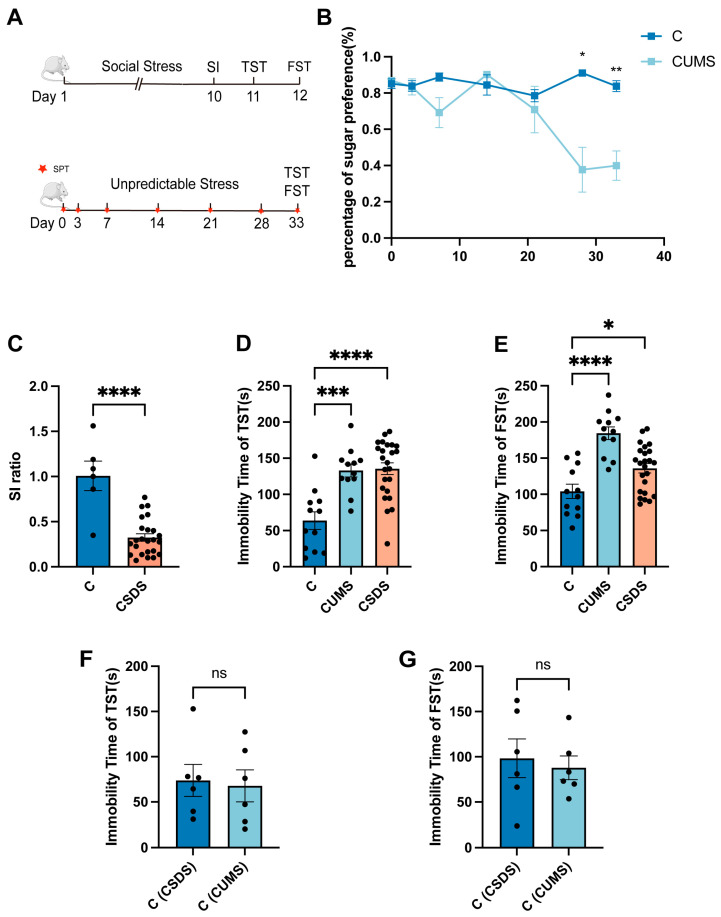
Two different kinds of animal depression models. The modeling schedule of CUMS and CSDS (**A**). During CUMS modeling, mice’s preference for syrup decreased with modeling time (**B**). The social interaction ratio in the SI test in CSDS mice when compared with healthy control group (**C**). The quantification of immobility time in mice in healthy control group, CUMS model group and CSDS model group in TST (**D**) and FST (**E**) tests. The quantification of immobility time in mice in healthy control group of two different models in TST (**F**) and FST (**G**) tests. Data represent the mean ± SEM. Unpaired *t*-test with equal variance. One-way ANOVA analysis of variance followed by Tukey’s multiple comparisons test. * *p* < 0.05; ** *p* < 0.01; *** *p* < 0.00; **** *p* < 0.0001. ns—not significant.

In this part, two different animal models were used to represent humans’ depressive phenotypes. One was CUMS, which is regarded as a model of mild depression caused by long-term unknown stress (Figure 1A); the other was CSDS, a kind of major depressive disorder caused by chronic social frustration (Figure 1A). 

Subsequently, in terms of the above-mentioned features of animal models, a series of animal behavior tests were used to evaluate the results of modeling. First, CUMS was evaluated through anhedonia using the sugar preference test. During modeling, we conducted tests on days 1, 3, 7, 14, 21, 28 and 33. The test results showed that the mice did not have an obvious preference for syrup on the 28th day of the test, indicating that the modeling was successful, and the results on the 33rd day further proved that, with the extension of the modeling, the anhedonia behavior of the mice intensified (Figure 1B). In addition, since the CSDS model is a modeling paradigm based on aggression, the challenged mice showed obvious social avoidance behaviors. For example, in the social interaction test, the mice in the model group showed avoidance and fear of CD-1, and their time spent in the central social area was significantly reduced in the second stage of the test. Therefore, the depression-like phenotype of the CSDS model mice was assessed using the social interaction test. On day 11 after modeling, we performed the social interaction test. Consistent with previous reports, mice with a social index (SI) < 1 were regarded depression-sensitive (Figure 1C), and their results were significantly different from those of the controls. This shows that the mice in the model group exhibited obvious social avoidance behavior and that the CSDS model is representative and available. Finally, both the CUMS and CSDS models induced desperate behavior in mice—that is, significantly increased immobility duration in the TST and FST tests compared with the controls. Our results also prove this feature. Our results show that mice in both models exhibited behavioral despair in both tests, especially in the TST, and, as the severity of the model intensified, the desperate behavior of the mice was more pronounced (Figure 1D,E). In conclusion, all the behavioral results show that our modeling was successful, and the mice in the model group showed obvious depression-like behaviors compared with those in the control group, such as anhedonia, social avoidance and despair.

In addition, it is worth noting that, due to the differences in the modeling cycle of the two models, there was a difference in the ages of the mice in the two models at the time of behavioral evaluation. Therefore, we also evaluated the performance of the healthy control groups of the two models using the TST and FST tests, and there was no statistically significant difference (Figure 1F,G). We believe that age had no significant effect on our subsequent analyses.

### 3.2. Analysis of Proteomic Data Using Principal Component Analysis

**Figure 2:**
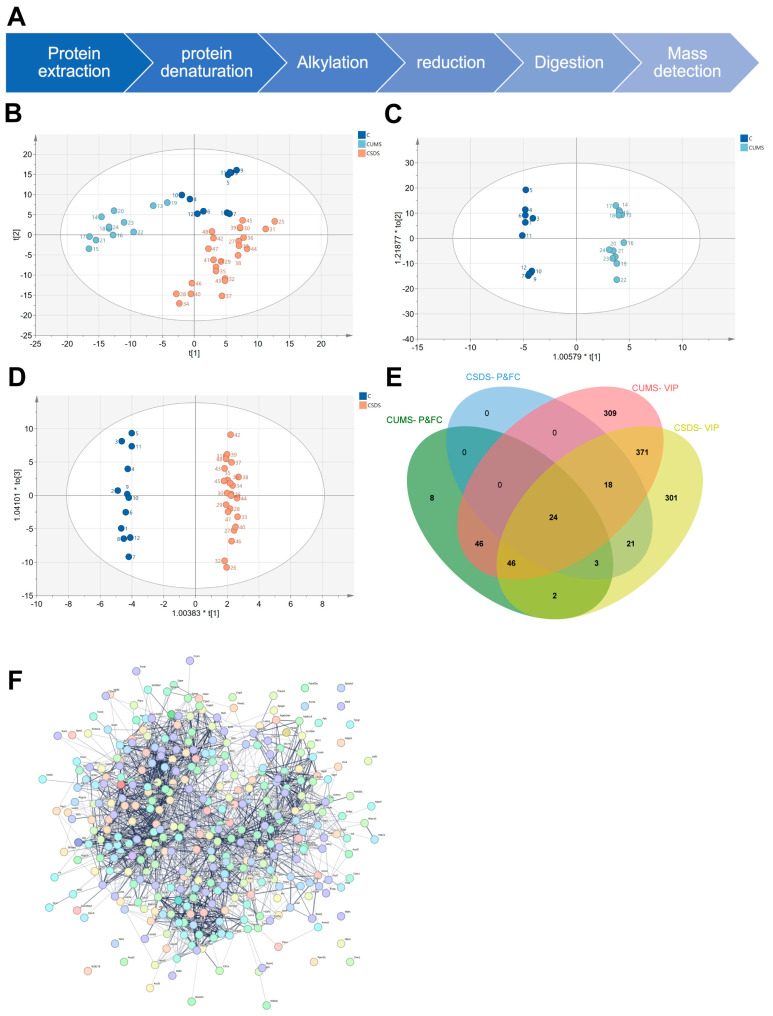
Analysis of proteomic data using principal component analysis. Untargeted proteomics workflow (**A**). PLS-DA analysis of DRN (**B**). OPLS-DA analysis of CUMS model and control group in DRN (**C**). OPLS-DA analysis of CSDS model and control group in DRN (**D**). With *p* < 0.05, fold change cutoff of 1.2 and VIP > 1 as the standard, the characteristic differential proteins of the CUMS model and the CSDS model were screened, and Venn diagrams were made for the characteristic differential proteins of the two models of DRN (**E**). A total of 684 proteins were screened for PPI network analysis (**F**).

The results of previous behavioral tests indicated that this model was successful in representing a subset of significant phenotypes in human depression. Therefore, we selected three brain regions (the hippocampus, DRN and mPFC) for untargeted proteomics detection based on reports in the related literature. After protein extraction, denaturation, alkylation, reduction, enzyme digestion and other processes, the protein was detected in the form of peptides via bottom-up non-targeted proteomics (Figure 2A). 

In the detection process, the IDA scanning mode was first used to establish the SWATH ion library. Subsequently, SWATH scanning was used to perform non-targeted proteomics detection, thereby reducing the impact of ion abundance on the detection results. Because, in the method setting, the width of the scanning window can be variable, it was set according to the density of the ion distribution (that is, the denser the ion distribution, the narrower the window) so as to maximize the accuracy of secondary quantitative information.

Subsequently, we applied the protein data matrix obtained using the SWATH method for principal component analysis. On the one hand, it was hoped that the overall change trend of the model histone protein compared with the healthy control group and the overall change characteristics of the protein between different models could be analyzed from an overall perspective.

First, PLS-DA analysis was performed on the three brain regions, respectively, using the SIMCA software. In the DRN brain region (Figure 2B), the characteristic distribution of the healthy control group was significantly different from that of the CUMS group and the CSDS group, while the CUMS and CSDS models had the same direction of change compared with the healthy control group. However, there were still significant differences between the two models. In addition, the hippocampus and mPFC brain regions were also characterized (Supplementary Figure S1A,D). However, the principal components used to distinguish different groups in the three brain regions were different, and the feature proteins in the three brain regions were also different. Our results show that, overall, the trends and regularities of the model changes among different brain regions were consistent. Additionally, there were differences between the different models. This shows that different depression models can effectively represent different aspects of depression. At the same time, it also proves the complexity of depression itself.

Furthermore, in order to obtain proteins that can distinguish the differences between different models and their control groups, we applied OPLS-DA to analyze each model and its healthy control group separately in each brain region (Figure 2 C,D, Supplementary Figure S1B,C,E,F). Subsequently, we screened the characteristic proteins that significantly contributed to the model discrimination, with a VIP value > 1, a *p* value < 0.05 and an FC cutoff value of 1.2 (Supporting Table S4). We screened three, twenty-four and six-hundred-fifty-seven differential proteins shared by the two models in the hippocampus, DRN and mPFC, respectively (Figure 2E, Supplementary Figure S1G,H). According to our results, the features of different brain regions were significantly different, which shows that the occurrence of depression has different degrees and different types of effect in each brain region. However, since there were no proteins with shared changes to enable us to determine associations between the different brain regions, we performed protein–protein interaction (PPI) analysis on the above 684 proteins in the online database of strings (Figure 2F). The results show that depression is mainly involved in alteration of the process of dopamine biosynthesis, the negative regulation of membrane tubes, the fusion of synaptic vesicles and the presynaptic active zone membrane, etc. 

### 3.3. Proteomics Biomarker Panels Construction

Proteomic analyses were performed in two depression models and in three depression-related brain regions. Different brain regions and different models possess different features. In very few cases, these features overlap, and, in more cases, it was difficult for us to find completely consistent characteristics. Our previous analysis mainly involved the extraction of potential features in a single model of a single brain region, but it was difficult to link the features of the three brain regions. Therefore, we further applied the AI technology of machine learning to jointly analyze the three brain regions and find a connection between their respective feature differences so as to better extract and screen features and distinguish the different depression models from the healthy controls.

**Figure 3:**
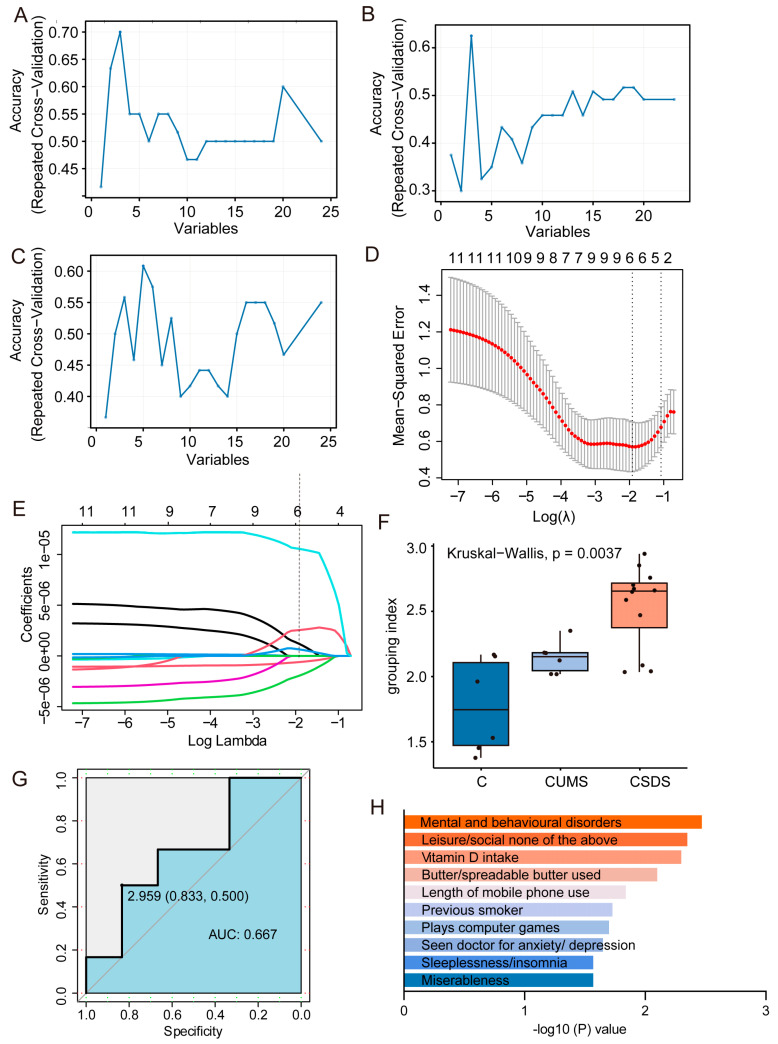
Proteomics biomarker panels construction. In hippocampus (**A**), DRN (**B**) and mPFC (**C**), the accuracy of machine learning varies with the number of feature proteins. Cross-validation plot for the penalty term (**D**). A linear combination of selected features’ respective coefficients (lambda = 0.1471366) (**E**) of box-and-whisker plot of grouping index (**F**). The ROC of established model (=2.302 − 9.625 × e−7 × AP2B1 × (−3.464)× e−6 × SRCN1 + 3.180 × e−6 × SYIM + 2.133 × e−6 × LSAMP + 1.253 e−6× CPNE6 + 1.125 × e−5 × EZRI) based on six characteristic proteins (**G**). Feature protein pathway enrichment results (**H**).

First, the exported protein peak area data matrix was randomly divided into two groups: a test group (*n* = 24) and a validation group (*n* = 24). We first used the caret package to separately select features for the hippocampus, DRN and mPFC brain regions. The results show that the prediction accuracy of the model was highest when the hippocampus (Figure 3A), DRN (Figure 3B) and mPFC (Figure 3C) had three, three and five independent variables, respectively. Then, all the feature proteins of the three brain regions were used for further (depression) protein selection using LASSO logistic regression algorithms, and the depression feature protein was generated via a linear combination of selected features weighted by their respective coefficients (Figure 3D,E). We successfully established a model based on six characteristic proteins, namely AP-2 complex subunit beta (AP2B1), SRC kinase signaling inhibitor 1 (SRCN1), Isoleucine–tRNA ligase, mitochondrial (SYIM), limbic-system-associated membrane protein (LSAMP), Copine-6 (CPNE6) and Ezrin (EZRI). In addition, we applied the established model to the validation set to evaluate the predictive accuracy of the model. The results of the box-and-whisker plots show that, upon applying the model, the validation set data could be well differentiated, with a *p* value of 0.0037 (Figure 3F). Additionally, the ROC results showed that the AUC value of the model was 0.667 (Figure 3G). In summary, both the ROC results and box-and-whisker plots demonstrate that the model is usable. Further, we applied two depression models again (see Supplementary Figure S2 for the modeling scheme and behavioral evaluation results). After excluding missing data, we established another validation group (*n* = 20) to evaluate our model (Supplementary Figure S3). Finally, we performed pathway enrichment analysis on these six proteins in the UK biobank database (Figure 3H). The enriched related pathways mainly include psychological and behavioral abnormalities, vitamin D intake and negative emotions, such as tension/anxiety/depression, insomnia/sleep disorders, etc. These terms directly indicate that our results are closely related to the incidence of depression. Additionally, these proteins are also strongly linked to psychiatric disorders. Moreover, we searched the UniProt database website for the functions of each of the six feature markers (Supporting Table S5). Additionally, the main biological functions involved were energy metabolism, nerve projection, synaptic function, selective neuronal growth and axon targeting, calcium-mediated intracellular processes, etc. These results are consistent with the PPI results of the two models sharing differential proteins in each brain region, obtained using the OPLS-DA analysis method.

In conclusion, the above experimental results suggest that machine learning analysis methods can indeed distinguish between groups with fewer feature markers than OPLS-DA. Moreover, the screened proteins are still closely related to the nervous system in function. 

### 3.4. Analysis and Verification of Expression Differences in Featured Proteins

Our research focuses on analyzing proteomic signature differences using artificial intelligence techniques to characterize depression models and find potentially powerful targets. In this section, we further analyze the relative expression of the characteristic proteins obtained from ML and verify their expression at the mRNA level. Then, our results are validated and proven to be replicable. 

**Figure 4:**
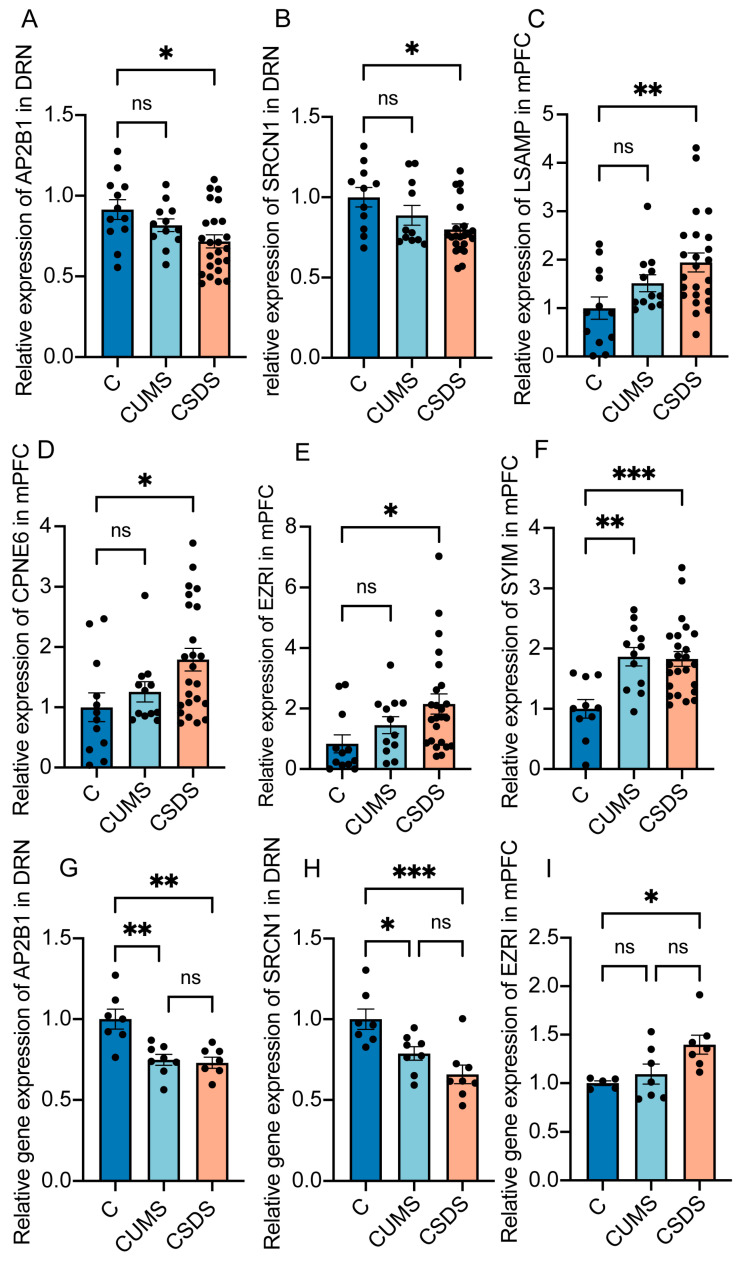
Analysis and verification of expression differences in featured proteins. Taking healthy control as the standard, after normalizing the peak area, the fold change of AP2B1 (**A**), SRCN1 (**B**), LSAMP (**C**), CPNE6 (**D**), EZRI (**E**) and SYIM (**F**). At the mRNA level, the fold change of AP2B1 (**G**), SRCN1 (**H**) and EZRI (**I**) in CUMS model and CSDS model compared with the control group. Data represent the mean ± SEM. One-way ANOVA analysis of variance followed by Tukey’s multiple comparisons test. * *p* < 0.05; ** *p* < 0.01; *** *p* < 0.001. ns—not significant.

First, we normalized the peak areas detected via proteomics and analyzed the fold changes of the six proteins in the different groups so that we could clearly determine the different trends of the changes in feature proteins. Additionally, regarding the brain regions used for comparison, we chose the region to be calculated via ML. Surprisingly, although the results of machine learning demonstrated that the screened feature proteins could significantly distinguish healthy controls in the CUMS and CSDS models, not every protein showed a significant difference in the fold change analysis (Figure 4A–F). Subsequently, we chose three of them and validated the differences at the mRNA level. The results show that AP2B1, SRCN1 in DRN and EZRI in mPFC varied significantly in the model group (Figure 4G–I). In addition, we chose two proteins, EF2 and SAHH2, which did not belong to the six proteins used for modeling but belonged to the eleven proteins obtained in the process of screening features by brain regions. Then, we tested their relative protein expressions and also verified their mRNA expressions. Although there were differences in SAHH2 (Supplementary Figure S4A,C) of the hippocampus at both the mRNA level and the protein level, the change trends of the two models were inversed. Additionally, regarding EF2 (Supplementary Figure S4B,D), the trend of its mRNA level was the inverse of that of its protein level. 

In conclusion, the above results once again demonstrate that machine learning screened feature markers that could significantly distinguish the model from the healthy control group. Additionally, the differential expression of these proteins is reproducible.

## 4. Discussion

First of all, whether it is the differential protein obtained by OPLS-DA analysis or the differential protein extracted by machine learning, the pathway enrichment results are related to synaptic connection and vesicle transport. This suggests that our different models of depression may all be related to abnormalities in synaptic connectivity and vesicle-transport-related functions. In addition, machine learning can efficiently screen out a relatively small number of characteristic proteins and has better discrimination for models. This suggests that these characteristic proteins may be highly correlated with diseases, and there are differences in the mRNA level of the screened proteins, indicating that our results are reproducible and reliable. Finally, it is worth considering the results of PLS-DA that the overall direction of protein changes in brain regions of CUMS depression and CSDS depression mice was not always in the same direction compared with healthy controls. It is suggested that our different depression models may have differences in pathogenesis and disease phenotype; at the same time, it may also be one of the reasons why clinical drug efficacy varies among patients with depression. 

Furthermore, the results of our screening were also validated in other studies. On the one hand, through gene database analysis, Feng et al. identified AP2B1 as a potential therapeutic target for major depressive disorder [26]. At the same time, fluoxetine treatment can significantly increase the expression of AP2B1 [27]. In addition, clinical studies by K Koido et al. have shown that LSAMP gene polymorphisms are closely related to major depression [28], and preclinical studies have also shown that the LSAMP gene plays an important role in regulating anxiety in rodents and that a lack of this gene can cause weakened adaptation in stressful environments [29]. Through the retrieval of protein functions, SYIM is closely related to the process of energy metabolism, including participating in ATP binding, etc. The research of Tianming Gao et al. proved that energy metabolism such as ATP is closely related to depression [30]. Furthermore, there are several reports stating that Copine 6 is decreased in depression animal models, which is also the antidepressant target of quercetin [31]. However, the obtained change direction of Copine 6 in depression in this study is contradictory to that in other reports, and further verification is needed. Although we failed to find the mechanism of EZRI in other reports in relation to depression, the proteomics report [32] by S S Newton et al. on the relationship between the choroid plexus (CP) and chronic stress also focused on this protein; thus, we consider EZRI as a powerful new target of depression. Finally, SRCN1 is a negative regulator of SRC, inhibiting SRC activity and downstream signaling through CSK. In the striatum of depressed rats, SRC kinase activity was enhanced [33], which also confirms our results. In conclusion, these four characteristic proteins may be closely related to the occurrence and development of depression and are powerful potential therapeutic targets.

In addition, considering the functions of proteins, we still believe that the proteins we found are important. The brain is a complex and precise organ. Hundreds of millions of neurons are connected with each other through synapses and precisely control all activity in the body. Affective disorders such as depression, in many cases, are due to abnormalities in the conduction of nerve signals in specific brain regions, which lead to emotional instability and behaviors such as anhedonia, despair and social avoidance. Among the targets we screened, AP2B1 is involved in the reuptake of synaptic signaling molecules. Its reduction in depression models appears to lead to an accumulation of signaling molecules in the synaptic cleft, which, in turn, leads to the excessive inhibition or activation of postsynaptic neurons. The function of SRCN1 is closely related to the release of neurotransmitters and the projection of neurons. At the same time, the main difference between the above two proteins exists in the DRN brain region, which further confirms the close relationship between nerve signal transduction disorder in the DRN brain region and depression. In addition, the functions of LSAMP, CPNE 6, SRCN1 and other proteins are all related to the growth and development of dendritic spines, which means that abnormal connection density between neurons may also lead to emotional instability and the development of emotional disorders. Overall, whether through functional analysis or comparison with other people’s research results, the differential proteins we found are indeed very important in depression. Additionally, our analysis method is reliable in terms of both its reproducibility and disease correlation.

Inevitably, our research also has certain shortcomings. Due to the limitation of long injection time for proteomics (90 min for one injection), our biological replicates were relatively small (*n* = 6–24). Therefore, individual differences had a greater impact on the results, leading to insignificant fold changes in characteristic proteins between different groups. Furthermore, our experiments were performed on mice, and, although C57BL/6J mice share more than 90% genetic similarity with humans, our results are not representative of clinical results. Therefore, the target still needs to be validated on clinical samples in the future.

## Data Availability

Data is contained within the article or supplementary material.

## Supplementary Materials

The following supporting information can be downloaded at: https://www.mdpi.com/article/10.3390/brainsci13050763/s1, Supporting Table S1. SWATH Variable Window Calculator_V1 in Excel Supporting Table S2. The window description file. Supporting Table S3. The protein peak area data matrix in excel format. Supporting Table S4. List of proteins obtained by conditional screening. Supporting Table S5. the functions of all feature markers in three different brain regions. Supporting Table S6. the summary of mouse brain ion library. Supplementary Figure S1 Analysis of proteomic data using principal component analysis. Supplementary Figure S2 Two different kinds of animal depression models. Supplementary Figure S3 The validation of the model. Supplementary Figure S4 Analysis and Verification of Expression Differences of Featured Proteins.
