# Supplementary material for: AI Machine Learning Technique Characterizes Potential Markers of Depression in Two Animal Models of Depression
Source: Brain Sci. 2023 May 5;13(5):763. doi: 10.3390/brainsci13050763 (PMC10216178; doi:10.3390/brainsci13050763)
Supplement: Supplementary file 1 [file brainsci-13-00763-s001.zip › supplimentary figure (1).pdf]

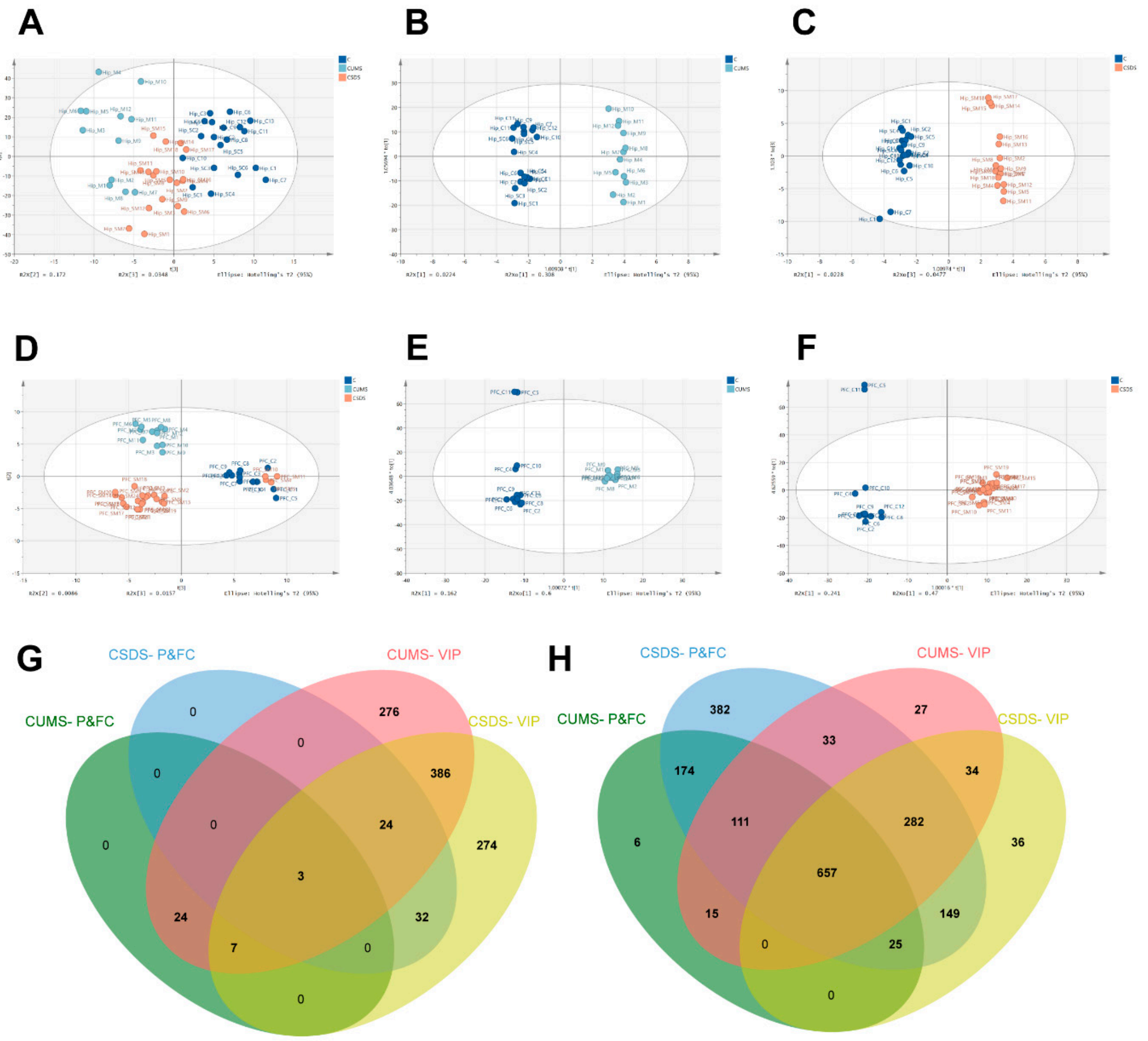

Supplementary Figure S1 Analysis of proteomic data using principal component analysis. PLS-DA analysis of Hippocampus (A), and mPFC (D). OPLS-DA analysis of CUMS model and its control group in Hippocampus (B), and mPFC (E). OPLS-DA analysis of CSDS model and its control group in Hippocampus (C), and mPFC (F). With P<0.05, Fold Change cut off 1.2, VIP>1 as the standard, the characteristic differential proteins of the CUMS model and the CSDS model were screened, and Venn diagrams were made for the characteristic differential proteins of the two models of Hippocampus (G), and mPFC (H), respectively.

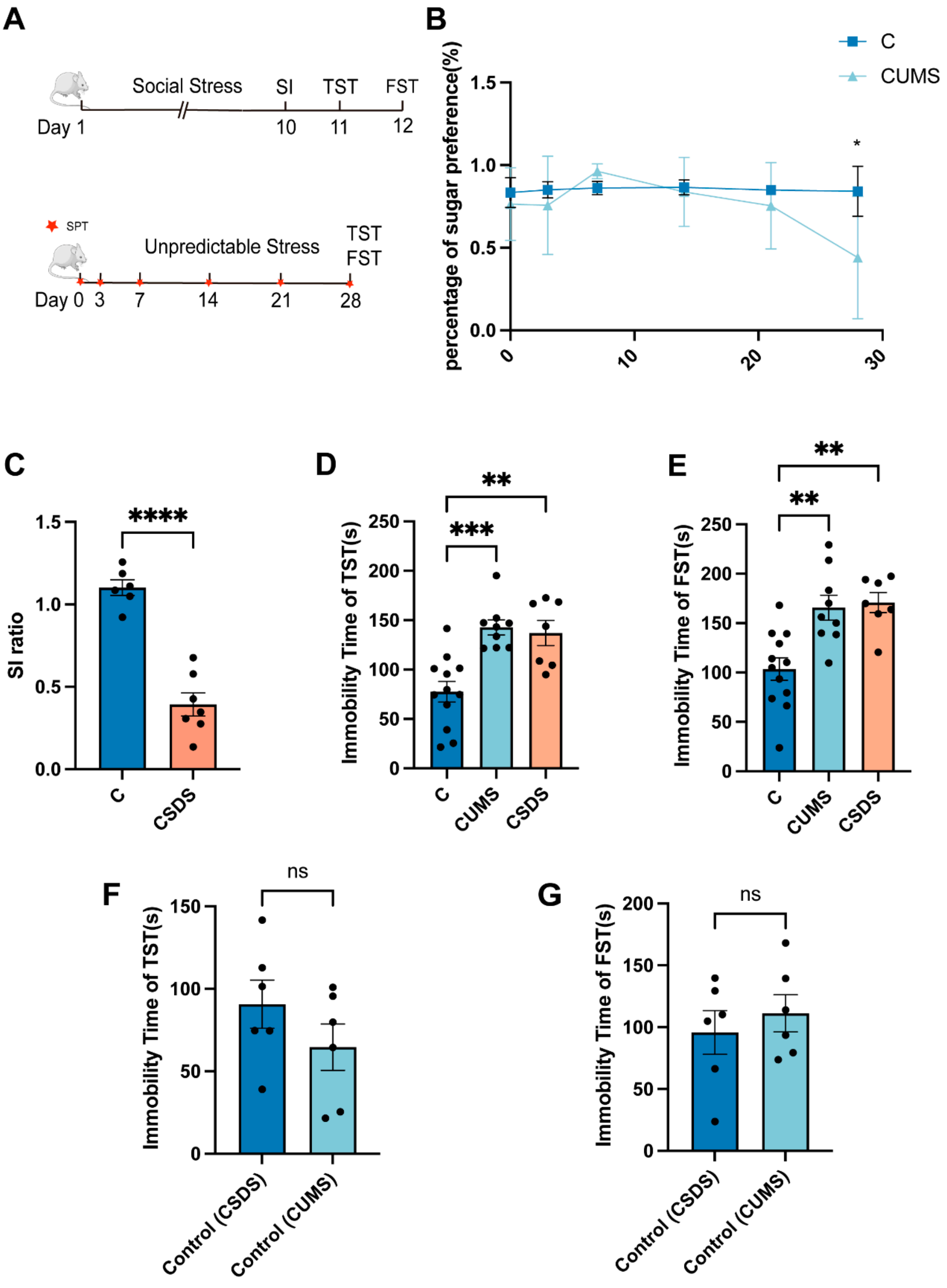

Supplementary Figure S2 Two different kinds of animal depression models. The modeling schedule of CUMS model (healthy control, n=6; CUMS model, n=7) and CSDS model (healthy control, n=6; CSDS model, n=7) (A). During CUMS modeling, mice's preference for syrup decreased with modeling time (B). The social interaction ratio in the SI test in CSDS mice when compared with healthy control group (C). The quantification of immobility time in mice in healthy control group, CUMS model group and CSDS model group in TST (D) and FST (E) tests. The quantification of immobility time in mice in healthy control group of two different models in TST (F) and FST (G) tests. Data represent the mean ± SEM. Unpaired t-test with equal variance; One-way ANOVA analysis of variance followed by Tukey's multiple comparisons test: * P< 0.05, ** P< 0.01, *** P< 0.00,; **** P< 0.0001, n.s. not significant.

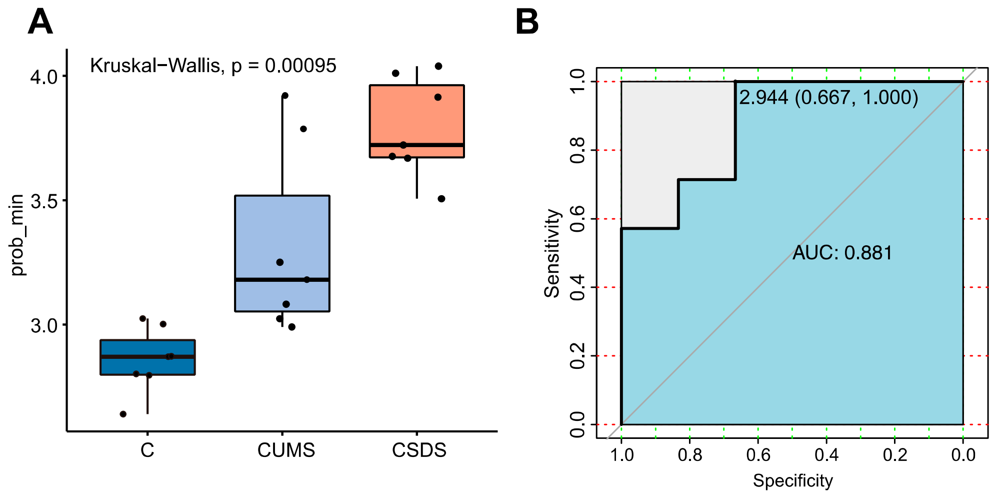

Supplementary Figure S3 The validation of the model. A box-and-whisker plot of grouping index (A). The ROC of established model based on six characteristic proteins (B)

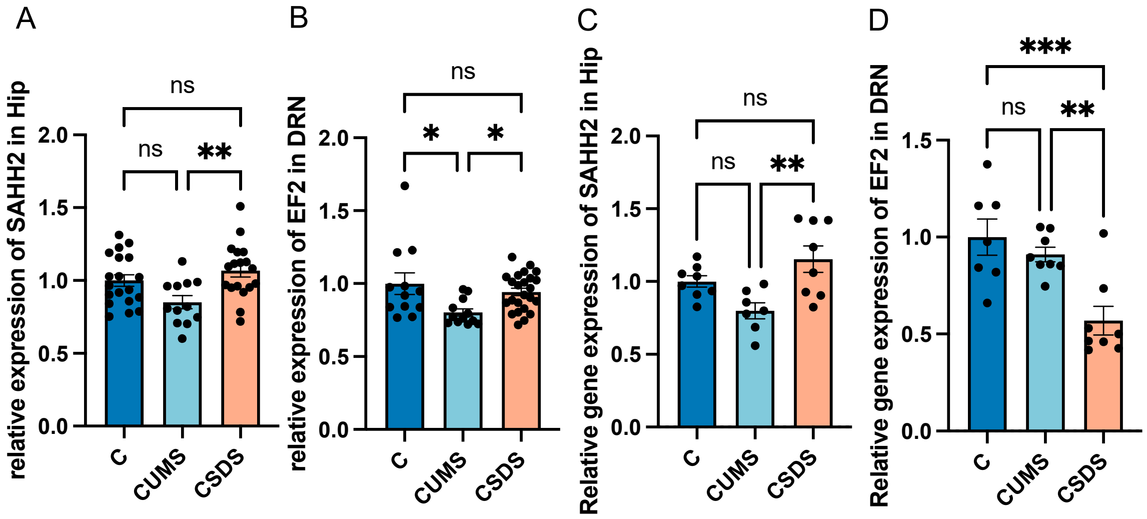

Supplementary Figure S4 Analysis and Verification of Expression Differences of Featured Proteins. Taking healthy control as the standard, after normalizing the peak area, the fold change of SAHH2 (A), and EF2 (B). At the mRNA level, the fold change of SAHH2 (C), and EF2 (D) in CUMS model and CSDS model compared with the control group. Data represent the mean ± SEM. One-way ANOVA analysis of variance followed by Tukey's multiple comparisons test, * $P< 0.05$; ** $P< 0.01$; *** $P< 0.001$, n.s., not significant.
