# Supplementary material for: AI Machine Learning Technique Characterizes Potential Markers of Depression in Two Animal Models of Depression
Source: Brain Sci. 2023 May 5;13(5):763. doi: 10.3390/brainsci13050763 (PMC10216178; doi:10.3390/brainsci13050763)
Supplement: Supplementary file 1 [file brainsci-13-00763-s001.zip › supporting information description.pdf]

AI technique of machine learning characterizes potential markers of depression in two models of depression

Zhang, Jing[1]; Zhang, Ran[1]; Peng, Ying[1]; Aa, Jiye[1*]; Wang, Guangji[1**]

1 Key Lab of Drug Metabolism & Pharmacokinetics, State Key Laboratory of Natural Medicines, China Pharmaceutical University, Tongjiaxiang 24, Nanjing 210009, P.R. China

Supporting Table S1. SWATH Variable Window Calculator_V1 in Excel

Supporting Table S2. The window description file.

Supporting Table S3. The protein peak area data matrix in excel format.

Supporting Table S4. List of proteins obtained by conditional screening.

Supporting Table S5. the functions of all feature markers in three different brain regions.

Supporting Table S6. the summary of mouse brain ion library.

Supplementary Figure S1 Analysis of proteomic data using principal component analysis.

Supplementary Figure S2 Two different kinds of animal depression models.

Supplementary Figure S3 The validation of the model.

Supplementary Figure S4 Analysis and Verification of Expression Differences of Featured Proteins.
